# CDAI trajectories in patients with rheumatoid arthritis treated with abatacept in real-world

**DOI:** 10.1186/s42358-026-00554-y

**Published:** 2026-06-23

**Authors:** Janet E. Pope, John S. Sampalis, Boulos Haraoui, Emmanouil Rampakakis, Dylan Keating, Fiona Allum, Marc-Olivier Trepanier, Louis Bessette

**Affiliations:** 1 Divisions of Rheumatology, Epidemiology, and Biostatistics, Department of Medicine, Western University, London, ON Canada; 2 JSS Medical Research, Inc., Montreal, QC Canada; 3 Faculty of Medicine, Department of Surgery, McGill University, Université de Montréal, Montreal, QC Canada; 4 Institut de Rhumatologie de Montréal, Montreal, QC Canada; 5 Department of Pediatrics, McGill University, Montreal, QC Canada; 6 Bristol Myers Squibb, Montreal, QC Canada; 7 Université de Laval, Centre Hospitalier de l’Université Laval, Québec, QC Canada; 8 Groupe de recherche en rhumatologie et maladies osseuses (GRMO), 100-1200 avenue Germain-des-Prés, Québec, QC G1V 3M7 Canada

**Keywords:** Rheumatoid arthritis, Abatacept, Clinical disease activity index, Trajectory-based

## Abstract

**Background:**

To describe the 12-month Clinical Disease Activity Index (CDAI) trajectory patterns and their determinants in patients with rheumatoid arthritis (RA) treated with subcutaneous abatacept.

**Methods:**

This was a post hoc analysis of adult patients with RA from the Abatacept Best Care (ABC) study. A growth mixture model (GMM) with Bayesian imputation for missing endpoints was used to define CDAI trajectory groups over 12 months. Determinants of trajectory patterns were identified among baseline patient and disease characteristics using bivariate comparisons and multivariable logistic regression with stepwise selection.

**Results:**

For the overall study sample (*n* = 256) mean (SD) age was 60.1 (11.5) years, 191 (75%) were female, and baseline CDAI was 30.1 (10.6). The GMM identified 3 trajectory groups that were classified as Rapid Responders (RR; *n* = 125 [49%]), Late Responders (LR; *n* = 96 [38%]), and Non-Responders (NR; *n* = 35 [14%]). RR had lower baseline CDAI, Disease Activity Score 28 - C-Reactive Protein, Patient Global Assessment (PtGA), Routine Assessment of Patient Index Data 3, Simplified Disease Activity Index, and Tender Joint Count compared to LR and NR (*p* < 0.001). Lower baseline PtGA, Rheumatic Disease Comorbidity Index, and lack of prior biologic use were significant predictors of RR (*p* < 0.03). CDAI scores at 3 months were not predictive of 12-month response to treatment.

**Conclusions:**

Approximately 85% of the abatacept-treated patients with RA in this study showed Rapid or Late CDAI reduction over 12 months. Trajectory-based analyses are informative and may have implications for clinical practice and research.

**Trial registration:**

This study was registered on 06 September 2017 in ClinicalTrials.gov under the registration number NCT03274141.

**Supplementary Information:**

The online version contains supplementary material available at https://doi.org/10.1186/s42358-026-00554-y.

## Background

Rheumatoid arthritis (RA) is an autoimmune disease characterized by chronic joint inflammation, which occurs in approximately 5 per 1000 adults [1]. Chronic inflammation of the synovial membrane can result in progressive cartilage and bone damage, impairing mobility [2] and contributing to long-term pain and disability. Current therapies include non-steroidal anti-inflammatory drugs (NSAIDs), glucocorticoids, and disease-modifying anti-rheumatic drugs (DMARDs) [3]. Unlike NSAIDs, DMARDs can improve symptoms of RA while also inhibiting structural joint damage, thereby helping prevent long-term disability [1]. 

Abatacept is a recombinant fusion protein that downregulates T cell activation by competing with CD28 for binding to CD80/CD86 [4]. Clinical and observational studies have shown that abatacept is effective at reducing RA symptom severity and improving patient outcomes [5–10]. However, response to DMARD treatment is heterogeneous [9, 11], and previous studies suggest that response to abatacept may vary according to clinical and disease characteristics such as prior treatments, disease severity, anti-citrullinated protein antibody status [12–14], and shared epitope status [15]. Identifying predictors of clinical response remains an important goal to reduce the risk of irreversible joint damage and improve long-term patient outcomes in patients with RA, yet there are currently no widely accepted predictive markers.

In routine clinical practice, treatment decisions are frequently guided by response to treatment at specific time points or reported mean/median time to response, however recent studies have shown that patient response may vary over time [11, 16, 17]. For example, Siemons et al. showed that disease activity in patients with RA was not linear in the first year of treatment [17]. Examining the trajectory of RA treatment over time may be more relevant for evaluating response to treatment, as this allows researchers to identify subgroups among patients with heterogeneous progression. These patient subgroups can then be evaluated for predictors of disease progression or response to therapy. Better understanding of disease trajectories and their associated determinants may support more realistic expectation setting and aid in treatment optimization by aligning healthcare professional and patient management decisions with likely response patterns rather than isolated assessments.

Growth mixture models (GMM) are a useful tool for investigating treatment response over time. Courvoisier et al. applied GMM to Disease Activity Score 28 (DAS28) trajectories after initiating abatacept and reported that drug retention varied depending on response trajectory [11]. Given that Clinical Disease Activity Index (CDAI) is more practical in the clinical setting than DAS28 in routine assessment of disease activity in patients with RA, the current study sought to identify patient subsets with distinct CDAI response trajectories over 12 months among patients treated with subcutaneous abatacept and to identify baseline factors associated with these trajectories.

## Methods

### Patients and study design

This was a post hoc analysis of data from 290 adult patients with RA (≥ 18 years of age) who were initiated on treatment with subcutaneous abatacept and enrolled in the Abatacept Best Care (ABC) study (NCT03274141) [3]. Patients had moderate to severely active RA (defined as CDAI > 10). Patients were excluded if they had received abatacept prior to the enrolment visit, failed more than 1 prior bDMARD therapy, or had a history of autoimmune disease or any joint inflammatory disease other than RA (except for concomitant secondary Sjögren’s syndrome).

The methodology of the ABC study has been reported elsewhere [3]. Briefly, treatment with abatacept was initiated as part of routine care as per the judgment of the treating physician prior to, and independently of, consideration for study enrollment. Physicians were randomized in a 1:1 ratio to either receive formal instruction on the Treat to Target (T2T) approach prior to the study or continue managing through routine care without formal training. All patients provided informed consent and fulfilled reimbursement criteria for treatment with subcutaneous abatacept under provincial or private health insurance coverage.

A minimum of two CDAI measurement time points were required to be included in the current analysis, one at baseline and at least one after baseline, resulting in a total of 256 of the 290 patients being included in this analysis.

### Follow-up and outcomes

Patients were assessed at baseline (0 months) and at 3, 6, 9, and 12 months. Disease activity was assessed using CDAI. The CDAI components are tender joint count (TJC), swollen joint count (SJC), physician global assessment (MDGA), and patient global assessment (PtGA), which were evaluated at each visit. CDAI scores range from 0 to 76, with low disease activity (LDA; including remission) and remission defined as CDAI ≤ 10 and ≤ 2.8, respectively. Additional baseline measures included Pain, Fatigue, Health Assessment Questionnaire-Disability Index (HAQ-DI), Routine Assessment of Patient Index Data 3 (RAPID3), DAS28, CDAI, Simple Disease Activity Index (SDAI), and Work Productivity and Activity Impairment (WPAI) scores.

### Statistical analyses

#### Growth mixture model

A growth mixture model (GMM) was used to identify CDAI 12-month trajectory groups. This method classified patients into otherwise unobservable subgroups or classes based on the changes in CDAI score over 12 months. To account for missing CDAI measurements, Bayesian data imputations replaced the missing data. Different regression models of increasing class numbers and degrees were tested and the model providing the best fit for the data was selected. The model defined the optimal number of classes and individual patient membership in these classes. More specifically, a baseline polynomial regression model was selected using Expected Log Pointwise Predictive Density (ELPD) by comparing polynomial degrees up to quartic. The baseline model assumed a single group for all the patient trajectories. A cubic model was selected because it best fit the data without overfitting. Next, GMM was used to determine if the data were consistent with multiple trajectory groups by comparing fits with multiple groups to the single group baseline model. A fully Bayesian approach, using Markov Chain Monte Carlo, was used to obtain the full model posterior distribution. The preferred number of groups was selected using the ELPD, and a 3-group model provided the best fit to the data without overfitting compared to the baseline model and the 2- and 4-group models. Each patient was assigned to one of the three trajectory groups (Responders, Late Responders, and Non-Responders) using the model posterior probability to select the group for each patient based on their CDAI trajectory. For each patient, the group with the highest probability of membership was selected.

#### Kaplan Meier curves

For each trajectory group, Kaplan Meier curves were generated for time to CDAI LDA/remission and remission and restricted mean survival times (RMST) were calculated. The log-rank test was used to assess the difference between the three trajectory groups with respect to the survival function for CDAI LDA/remission and remission.

#### Baseline characteristics comparisons

Baseline patient and disease characteristics were summarized by trajectory group. Statistical significance for the differences between the trajectory groups with respect to these characteristics was determined using Pearson’s Chi-squared test for categorical variables and One-way ANOVA for continuous variables.

#### Baseline predictors of response

To determine if baseline characteristics were predictive of CDAI response, patients were separated into two classes: Responders (Rapid Responders and Late Responders) and Non-Responders, as defined by CDAI trajectory input to a GMM using the method described above. Univariate logistic regression models with each baseline characteristic as the independent variable were used to identify potentially significant predictors using a cut-off of *p* < 0.15 [18]. 

Using the subset of predictors identified in the univariate analysis, multivariable logistic regression using forward- and backward-stepwise selection was used to identify baseline characteristics that were independently associated with a patient being a Responder. Primary Factor Analysis was used to identify clusters of variables that were highly correlated. Separate multivariable logistic regression models with stepwise selection were performed, including the variables of each factor. The final multivariable logistic regression model included only those predictor variables that were retained by the stepwise processes. A final stepwise selection was used on the final model to identify independent predictors of being a Responder.

#### Predictions at 3 months

In order to evaluate if the change in the first 3-months in CDAI measurements were predictive of the 12-month trajectory groupings a GMM was used to identify trajectory groupings based solely on the baseline and 3-month measurements. The patient classifications based on the three-month trajectories were then compared to the classification obtained with the full 12-month trajectories.

#### CDAI component analysis

The association between changes in the individual CDAI components over the 12-month period and CDAI trajectory classification was tested using Mixed Effects Repeated Measures Models.

GMM was also applied to the individual CDAI components to determine if these were sufficient to group patient trajectories without using the full composite score. The same approach used for the CDAI score was used for the individual components. The comparison between CDAI trajectory groups and component trajectory groups was performed using merged Rapid and Late CDAI Responders into a single CDAI Responder group.

Analyses were performed using the PyMC probabilistic programming library and SPSS version 28.

## Results

### 12-month trajectory groups

**Fig. 1:**
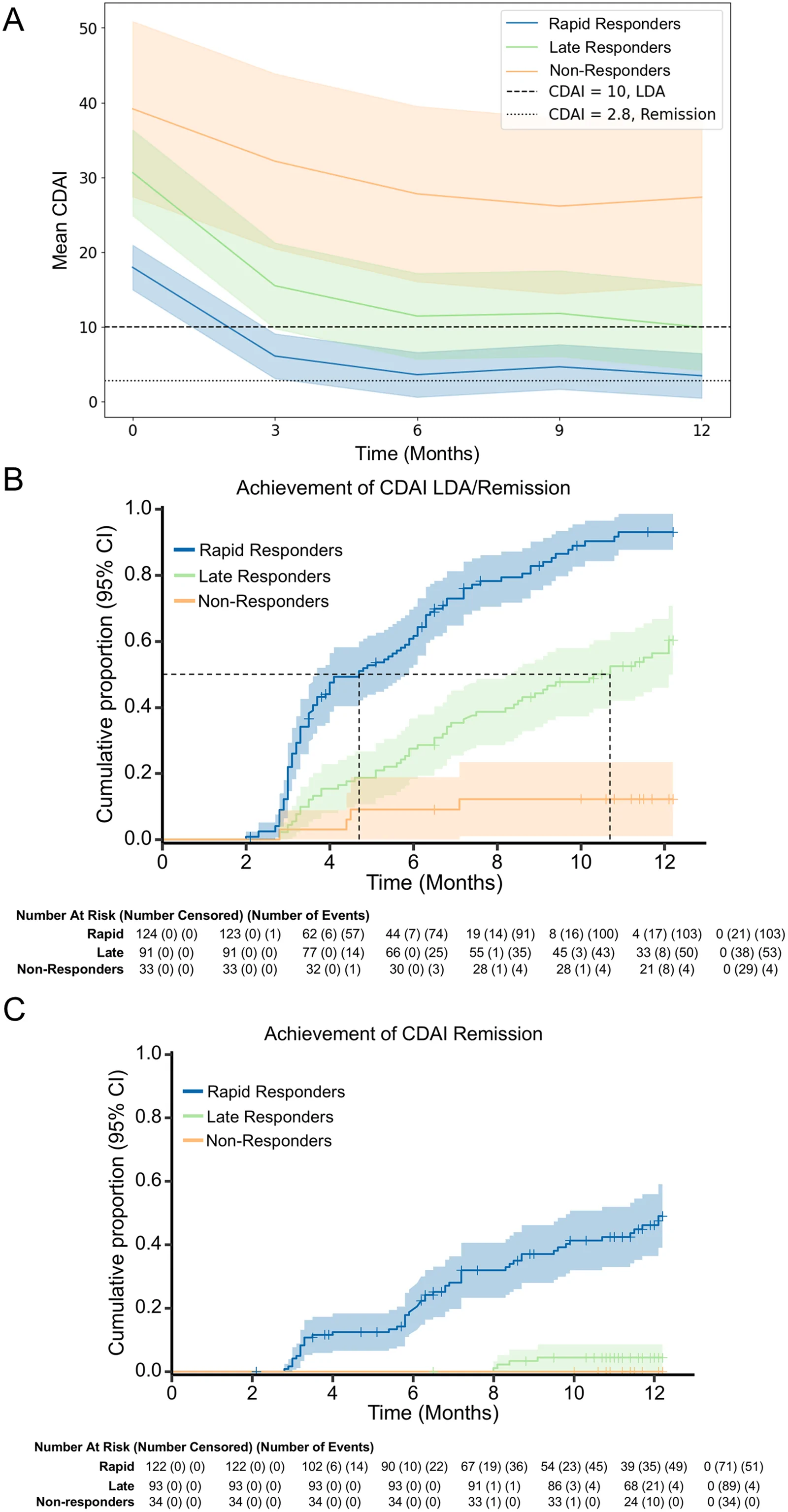
CDAI trajectory groups in patients with RA treated with abatacept over 12 months. **A**. Mean CDAI scores and standard deviation for the 12-month response trajectory groups for patients with RA treated with abatacept identified by GMM based on CDAI response over time. **B**. Inverse Kaplan Meier curves for time to low disease activity for the 3 CDAI trajectory groups over 12 months. Log-rank test: *p* < 0.001. **C**. Inverse Kaplan Meier curves for time to remission for the 3 CDAI trajectory groups over 12 months. Log-rank test: *p* < 0.001. CDAI: Clinical Disease Activity Index; CI: confidence interval; LDA: low disease activity

This analysis included 256 patients treated with abatacept, with a mean (SD) age of 60.1 (11.5) years, 191 patients (74.6%) were female, and the mean (SD) baseline CDAI score was 30.1 (10.6). The GMM analysis identified three CDAI trajectory groups: Rapid Responders (*n* = 125 [49%]), Late Responders (*n* = 96 [37%]), and Non-Responders (*n* = 35 [14%]) (Fig. 1a). The rates of achieving CDAI LDA (including remission) and remission were significantly different among the 3 groups (Fig. 1b and c; log-rank test *p* < 0.001 for both CDAI LDA/remission and remission). Median (95% CI) time to CDAI LDA/remission was 4.7 (3.7–5.7) months for Rapid Responders, 10.7 (8.4–12.1) for Late Responders and not achieved for Non-Responders. RMST (95% CI) to CDAI LDA/remission was 5.6 (5.1–6.1) months, 9.0 (8.2–9.7) months, and 11.3 (10.4–12.1) months for Rapid Responders, Late Responders and Non-Responders, respectively. RMST (95% CI) for time to CDAI remission was 9.6 (9.0–10.2) months for Rapid Responders and 12.0 (11.8–12.2) for Late Responders, while none of the Non-Responder patients reached CDAI remission within the 12-month follow-up period.

### Baseline characteristics of the three 12-month trajectory groups

The results in Table 1 show that the three patient groups were not different with respect to age, sex, and race. However, the Late Responder group had nominally higher mean BMI when compared to the other two groups (*p* = 0.083) and the mean Rheumatic Disease Comorbidity Index (RDCI) score was higher for the Non-Responder patients (*p* = 0.075). Baseline mean TJC, SJC, PtGA, MDGA, Pain, Fatigue, HAQ-DI, RAPID3, DAS28, CDAI, SDAI, as well as WPAI scores, were lower for the Rapid Responder group (*p* ≤ 0.02). Non-Responders had a higher proportion of involvement at baseline of the shoulder (*p* = 0.019), the knee (*p* = 0.133) and the elbow (*p* = 0.025) joints. Anti-cyclic citrullinated peptide (ACCP) antibody positivity was reported by a lower proportion of Non-Responder patients (*p* = 0.05). While morning stiffness was reported by the vast majority of patients, it was less in the Rapid Responder group when compared to Late and Non-Responders (*p* = 0.045) with a nominally higher duration of morning stiffness being reported by the Non-Responder patients (*p* = 0.103). In addition, Rapid and Late Responder patients were less likely to have had previous bDMARD treatment compared to Non-Responders (*p* < 0.001).

**Table 1:** Patient demographics and baseline characteristics

	CDAI Trajectory Group			
	Rapid Responders	Late Responders	Non-Responders	
	*N* = 125	*N* = 96	*N* = 35	*P* - Value
**Planned Treatment at Baseline**				
Routine Care	68 (54.4%)	60 (62.5%)	20 (57.1%)	0.480
Treat to Target	57 (45.6%)	36 (37.5%)	15 (42.9%)	
**Demographics**				
Age, years	59.44 (12.04)	61.29 (10.51)	59.15 (12.02)	0.435
Gender				
Female	90 (72.0%)	72 (75.0%)	29 (82.9%)	0.424
Male	35 (28.0%)	24 (25.0%)	6 (17.1%)	
BMI, kg/m^2^	28.35 (7.63)^a^	30.64 (7.01)^b^	28.63 (7.94)^c^	0.083
Race				
Caucasian	118 (94.4%)	90 (93.8%)	34 (97.1%)	0.856
Black	1 (0.8%)	0 (0.0%)	0 (0.0%)	
Asian	2 (1.6%)	2 (2.1%)	1 (2.9%)	
Hispanic	2 (1.6%)	1 (1.0%)	0 (0.0%)	
Native/Aboriginal	1 (0.8%)	0 (0.0%)	0 (0.0%)	
Other	1 (0.8%)	3 (3.1%)	0 (0.0%)	
Smoking Status				
Current	20 (16.1%)	17 (17.7%)	10 (28.6%)	0.453
Past	48 (38.7%)	34 (35.4%)	9 (25.7%)	
None	56 (45.2%)	45 (46.9%)	16 (45.7%)	
**Baseline RA Parameters**				
Rheumatic Disease Comorbidity Index [0–9]	1.5 (1.53)	1.72 (1.57)	2.17 (1.50)	0.075
RA Disease Duration, years	7.43 (8.61)	7.01 (8.66)	9.73 (11.07)	0.301
Known RF – Positive	74 (59.2%)	51 (53.1%)	16 (45.7%)	0.325
ACCP – Positive	45 (36.0%)	31 (32.3%)	5 (14.3%)	0.050
CRP, mg/dl	1.75 (2.86)^d^	2.51 (4.91)^e^	1.98 (2.32)^f^	0.367
ESR, mm/hr	21.78 (19.19)^g^	24.55 (20.12)^h^	18.83 (16.23)^i^	0.403
TJC [0–28]	7.94 (5.62)^j^	9.83 (5.48)^k^	15.20 (5.93)	< 0.001
SJC [0–28]	7.60 (4.32)	8.08 (4.47)	10.26 (5.50)	0.010
Tender or Swollen Shoulder	49 (39.2%)	41 (42.7%)	23 (65.7%)	0.019
Tender or Swollen Knee	57 (45.6%)	42 (43.8%)	22 (62.9%)	0.133
Tender or Swollen Elbow	31 (24.8%)	29 (30.2%)	17 (48.6%)	0.025
Tender or Swollen Wrist	90 (72.0%)	72 (75.0%)	25 (71.4%)	0.860
Tender or Swollen Hand	121 (96.8%)	92 (95.8%)	34 (97.1%)	0.904
Morning Stiffness				
No	15 (12.0%)	3 (3.1%)	2 (5.7%)	0.045
Yes	110 (88.0%)	93 (96.9%)	33 (94.3%)	
Morning Stiffness Duration				
< 30 min	30 (27.3%)^d^	24 (25.8%)^l^	5 (15.2%)^c^	0.103
30–60 min	31 (28.2%)^d^	27 (29.0%)^l^	4 (12.1%)^c^	
1–2 h	19 (17.3%)^d^	22 (23.7%)^l^	11 (33.3%)^c^	
> 4 h	30 (27.3%)^d^	20 (21.5%)^l^	13 (39.4%)^c^	
PtGA [VAS 0-100]	57.38 (22.91)^m^	67.25 (18.50)^n^	73.94 (18.62)	< 0.001
MDGA [VAS 0-100]	60.39 (17.65)^o^	61.22 (14.63)	71.09 (18.00)	0.003
Pain Assessment [VAS 0-100]	60.68 (25.81)^m^	64.94 (19.93)^n^	73.97 (21.93)	0.011
Fatigue Assessment [VAS 0-100]	58.51 (23.52)^m^	63.34 (22.54)^k^	76.50 (21.58)^p^	< 0.001
HAQ-DI [0–3]	1.35 (0.65)^m^	1.61 (0.60)^n^	1.63 (0.59)	0.005
RAPID3 [0–30]	14.08 (5.41)^j^	16.88 (4.53)^k^	17.85 (5.41)	< 0.001
DAS28-CRP	4.13 (0.99)	4.54 (0.85)	5.22 (0.74)	< 0.001
DAS28-ESR	4.43 (1.29)	4.89 (1.22)	5.17 (1.20)	0.002
CDAI [0–76]	26.97 (10.18)	30.49 (8.87)	39.96 (10.40)	< 0.001
SDAI [0-100]	28.56 (10.84)^j^	32.61 (10.94)	41.49 (10.43)	< 0.001
WPAI Absenteeism [VAS 0-100]	7.11 (21.33)^q^	12.18 (21.35)^f^	20.52 (30.17)^r^	0.164
WPAI Presenteeism [VAS 0-100]	42.49 (27.84)^s^	51.75 (27.40)^t^	58.69 (21.07)^u^	0.090
WPAI Work Productivity [VAS 0-100]	43.62 (28.86)^q^	52.29 (30.21)^f^	62.65 (21.83)^r^	0.088
WPAI Activity Impairment [VAS 0-100]	51.55 (26.56)^v^	58.07 (25.85)^w^	68.82 (19.99)^p^	0.002
**Prior Treatment for RA**				
Methotrexate	82 (70.1%)^v^	65 (73.9%)^x^	19 (57.6%)^c^	0.220
Corticosteroids	30 (25.6%)^v^	21 (23.9%)^x^	10 (30.3%)^c^	0.770
NSAIDS	40 (34.2%)^v^	25 (28.4%)^x^	11 (33.3%)^c^	0.668
Hydroxychloroquine	54 (46.2%)^v^	34 (38.6%)^x^	16 (48.5%)^c^	0.470
Sulfasalazine	20 (17.1%)^v^	14 (15.9%)^x^	9 (27.3%)^c^	0.326
Leflunomide	13 (11.1%)^v^	16 (18.2%)^x^	3 (9.1%)^c^	0.249
Gold salts	2 (1.7%)^v^	0 (0.0%)	0 (0.0%)	0.352
bDMARD	37 (29.6%)	46 (47.9%)	21 (60.0%)	< 0.001

Data shown are mean (SD) or N (%). ^a^*n*=114; ^b^*n*=91; ^c^*n*=33; ^d^*n*=110; ^e^*n*=81; ^f^*n*=27; ^g^*n*=99; ^h^=75; ^i^*n*=23; ^j^*n*=124; ^k^*n*=94; ^l^*n*=93; ^m^*n*=123; ^n^*n*=95; ^o^*n*=121; ^p^*n* = 34; ^q^*n*=53; ^r^*n*=12; ^s^*n*=55; ^t^*n*=32; ^u^*n*=13; ^v^*n*=117; ^w^*n*=89; ^x^*n*=88

*P*-values determined using Pearson’s Chi-2 test for categorical variables and the One-way ANOVA test for continuous variables

ACCP: anti-cyclic citrullinated peptide; ANOVA: analysis of variance; BMI: body mass index; CDAI: Clinical Disease Activity Index; CRP: C-Reactive Protein; DAS28: Disease Activity Score 28; DMARD: Disease-Modifying Antirheumatic Drugs; ESR: Erythrocyte Sedimentation Rate; HAQ-DI: Health Assessment Questionnaire-Disability Index; MDGA: Physician Global Assessment; PtGA: Patient Global Assessment; RA: Rheumatoid arthritis; RAPID3: Routine Assessment of Patient Index Data 3; RDCI: Rheumatic Disease Comorbidity Index; RF: Rheumatoid Factor; SDAI: Simplified Disease Activity Index for RA; SJC: Swollen Joint Count; TJC: Tender Joint Count; WPAI: Work Productivity and Activity Impairment

### Baseline predictors of 12-month CDAI trajectory

The results of the simple logistic regression analyses assessing the association between demographic and baseline parameters with the odds of being a Responder, defined as being classified in the Rapid Responder or Late Responder trajectory groups, are presented in Table 2. These results showed that lower baseline comorbidity as measured by the rheumatic disease comorbidity index (RDCI; odds ratio [OR] = 0.800; *p* = 0.044), anti-cyclic citrullinated peptide (ACCP) positivity (OR = 3.145; *p* = 0.023), lower TJC (OR = 0.836; *p* < 0.001), lower SJC (OR = 0.897; *p* = 0.004), and lower duration of morning stiffness (OR = 0.345; *p* = 0.066) were associated with increased odds of being a Responder. Lower odds of being in the Responder group were associated with baseline involvement of the shoulder (OR = 0.358; *p* = 0.007), knee (OR = 0.48; *p* = 0.05), and elbow (OR = 0.395; *p* = 0.012). Higher odds of being a Responder group were associated with lower baseline disease severity as measured by the PtGA (OR = 0.969; *p* = 0.002), MDGA (OR = 0.961; *p* = 0.001), RAPID3 (OR = 0.907; *p* = 0.009), DAS28-CRP (OR = 0.304; *p* < 0.001); DAS28 Erythrocyte Sedimentation Rate (ESR; OR = 0.707; *p* = 0.022), CDAI (OR = 0.901; *p* < 0.001), and SDAI (OR = 0.924; *p* < 0.001). Lower baseline scores in the WPAI Productivity (OR = 0.979; *p* = 0.080) and WPAI Impairment (OR = 0.975; *p* = 0.004) were associated with higher odds of being in the Responder groups. Prior treatment with a bDMARD was also associated with lower odds of being a Responder (OR = 0.401; *p* = 0.014).

**Table 2:** Simple logistic regression for predictors of response (Rapid Responder or Late Responder)

Variable	Odds ratio			*P*-Value
	Estimate	Lower 95% CI	Upper 95% CI	
**Planned Treatment**	1.032	0.502	2.122	0.931
**Demographics**				
Age	1.008	0.978	1.040	0.598
Gender (Female vs. Male)	1.760	0.696	4.453	0.232
BMI	1.014	0.962	1.069	0.600
Race				1.000
Black vs. Caucasian	NC			
Asian vs. Caucasian	0.654	0.071	6.027	0.708
Hispanic vs. Caucasian	NC			
Native / Aboriginal vs. Caucasian	NC			
Other vs. Caucasian	NC			
Smoking Status				0.193
Current vs. Never	0.586	0.244	1.406	0.232
Past vs. Never	1.443	0.606	3.435	0.407
**Baseline Disease Parameters**				
**Rheumatic Disease Comorbidity Index**	**0.800**	**0.643**	**0.994**	**0.044**
RA Disease Duration	0.974	0.941	1.008	0.135
RF (Positive vs. Negative / Unknown)	1.546	0.755	3.165	0.233
**ACCP (Positive vs. Negative / Unknown)**	**3.145**	**1.173**	**8.435**	**0.023**
CRP	1.007	0.899	1.128	0.907
ESR	1.013	0.987	1.040	0.333
**TJC**	**0.836**	**0.782**	**0.894**	**< 0.001**
**SJC**	**0.897**	**0.832**	**0.967**	**0.004**
**Tender or Swollen Shoulder (Yes vs. No)**	**0.358**	**0.170**	**0.757**	**0.007**
**Tender or Swollen Knee (Yes vs. No)**	**0.480**	**0.230**	**1.000**	**0.050**
**Tender or Swollen Elbow (Yes vs. No)**	**0.395**	**0.191**	**0.816**	**0.012**
Tender or Swollen Wrist (Yes vs. No)	1.098	0.498	2.424	0.816
Tender or Swollen Hand (Yes vs. No)	0.783	0.095	6.460	0.820
Morning Stiffness (Yes vs. No)	0.684	0.152	3.083	0.621
**Morning Stiffness Duration**				**0.042**
30–60 min vs. < 30 min	1.343	0.343	5.263	0.673
1–2 h vs. < 0.30 min	0.345	0.111	1.071	0.066
> 4 h vs. < 30 min	0.356	0.118	1.071	0.066
**PtGA**	**0.969**	**0.950**	**0.989**	**0.002**
**MDGA**	**0.961**	**0.938**	**0.984**	**0.001**
**Pain Assessment**	**0.975**	**0.957**	**0.994**	**0.009**
**Fatigue Assessment**	**0.964**	**0.944**	**0.984**	**< 0.001**
HAQ-DI	0.658	0.367	1.182	0.162
**RAPID3**	**0.907**	**0.843**	**0.976**	**0.009**
**DAS28-CRP**	**0.304**	**0.187**	**0.495**	**< 0.001**
**DAS28-ESR**	**0.707**	**0.526**	**0.951**	**0.022**
**CDAI**	**0.901**	**0.868**	**0.936**	**< 0.001**
**SDAI**	**0.924**	**0.894**	**0.954**	**< 0.001**
WPAI Absenteeism	0.983	0.963	1.004	0.115
WPAI Presenteeism	0.982	0.959	1.005	0.121
WPAI Work Productivity	0.979	0.956	1.003	0.080
**WPAI Activity Impairment**	**0.975**	**0.959**	**0.992**	**0.004**
**Prior Treatments for RA (Yes vs. No)**				
Methotrexate	1.868	0.878	3.970	0.105
Corticosteroids	0.762	0.340	1.707	0.509
NSAIDS	0.929	0.425	2.028	0.853
Hydroxychloroquine	0.799	0.383	1.669	0.551
Sulfasalazine	0.530	0.227	1.240	0.143
Leflunomide	1.648	0.472	5.752	0.434
Gold	NC			0.999
**Biologic DMARD**	**0.401**	**0.193**	**0.831**	**0.014**

ACCP: anti-cyclic citrullinated peptide; BMI: body mass index; CDAI: Clinical Disease Activity Index; CI: confidence interval; CRP: C-Reactive Protein; DAS28: Disease Activity Score 28; DMARD: Disease-Modifying Antirheumatic Drugs; ESR: Erythrocyte Sedimentation Rate; HAQ-DI: Health Assessment Questionnaire-Disability Index; MDGA: Physician Global Assessment; PtGA: Patient Global Assessment; NC: not calculated; NSAID: nonsteroidal anti-inflammatory drug; RA: Rheumatoid arthritis; RAPID3: Routine Assessment of Patient Index Data 3; RDCI: Rheumatic Disease Comorbidity Index; RF: Rheumatoid Factor; SDAI: Simplified Disease Activity Index for RA; SJC: Swollen Joint Count; TJC: Tender Joint Count; WPAI: Work Productivity and Activity Impairment

Development of the multivariable logistic regression model began with a full model including 19 predictors with clinically important or statistically noteworthy associations in the simple logistic regression analysis. However, due to significant multicollinearity, this model could not converge, and the results were not interpretable. Primary factor analysis was used to identify clusters of variables that were highly correlated. The factor analysis identified five factors representing the 19 variables. Five separate multivariable logistic regression models with stepwise selection were performed including the variables in each factor. The final multivariable logistic regression model included only those predictor variables that were retained by the five stepwise processes. A final stepwise selection was used on the final model to identify independent predictors of being a Responder (Table 3). The following baseline variables are independent predictors of being a Responder (Rapid Responder or Late Responder) over 12 months of treatment with abatacept: MDGA (OR = 0.972; *p* = 0.053), PtGA (OR = 0.975; *p* = 0.028), SJC (OR = 0.919; *p* = 0.05), and RDCI (OR = 0.751; *p* = 0.019) as well as prior treatment with bDMARD (OR = 0.358; *p* = 0.011).

**Table 3:** Final multivariate logistic regression for predictors of response (Rapid Responder or Late Responder)

Baseline variable	Odds ratio			
	Estimate	Lower 95% CI	Upper 95% CI	*P* - Value
MDGA	0.972	0.945	1.000	0.053
PtGA	0.975	0.953	0.997	0.028
SJC	0.919	0.844	1.000	0.050
Rheumatic Disease Comorbidity Index	0.751	0.591	0.954	0.019
Prior Biologic DMARD (Yes vs. No)	0.358	0.162	0.792	0.011

CI: confidence interval; DMARD: Disease-Modifying Antirheumatic Drugs; MDGA: Physician Global Assessment; PtGA: Patient Global Assessment; SJC: swollen joint count

### Predicting 12-month trajectories using 0- and 3-month CDAI scores

The GMM method with three-group classification was applied to the CDAI scores during the first three months of treatment. This analysis identified 118 (46.3%) Rapid Responders, 72 (28.2%) Late Responders and 65 (25.5%) Non-Responders during the first three months of treatment. The results in Table 4 show that the overall weighted kappa for the 3-month and 12-month CDAI trajectory classification was 0.47 (95% CI: 0.37–0.56) with an overall percent agreement of 63.6%. These results suggest the 3-month CDAI trajectory is not a valid predictor of the 12-month trajectory. In fact, of the 118 patients classified as Rapid Responders at 3 months, 27% were classified as Late Responders or Non-Responders at 12 months; of the 72 patients classified as Late Responders at 3 months, 30.5% were classified as Rapid Responders and 4.2% as Non-Responders at 12 months; and finally of the 65 patients classified as Non-Responders at 3 months, 24.6% were classified as Rapid Responders at 12 months (Table 4).

**Table 4:** Comparison of 3- and 12-month CDAI classifications

		Three-month trajectory classification*			Total
		Rapid responder	Late responder	Non-responder	
12-Month Trajectory Classification	Rapid Responder	86	22	16	124
	Late Responder	29	47	20	96
	Non-Responder	3	3	29	35
Total		118	72	65	255

Data are N. *3-month classification was not possible for one patient. Weighted kappa: 0.47 (95% CI: 0.37–0.56); overall percent agreement: 63.6%. CDAI: clinical disease activity index

### Change in CDAI components by CDAI trajectory group

**Fig. 2:**
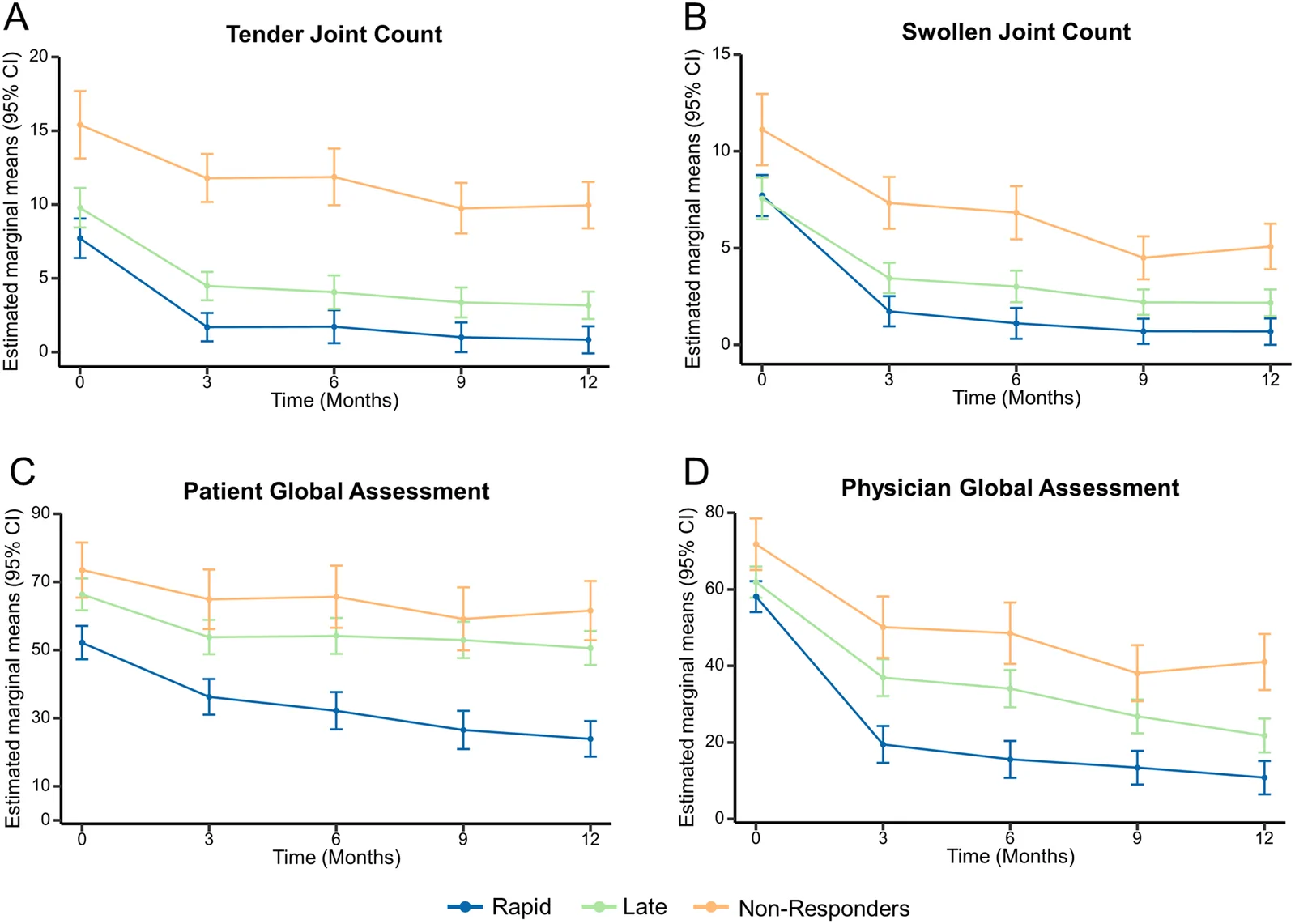
Repeated measures mixed effects marginal means of CDAI components, (**A**) Tender Joint Count, (**B**) Swollen Joint Count, (**C**) Patient Global Assessment, and (**D**) Physician Global Assessment, for the 3 CDAI Trajectory Groups Through 12 Months. Displayed are estimated marginal means and 95% confidence intervals. CDAI: Clinical Disease Activity Index; CI: confidence interval

The association between changes in the individual CDAI components over the 12-month period and CDAI trajectory classification was tested using Mixed Effects Repeated Measures Models. The change in all four CDAI components over time was statistically significant for all CDAI trajectory groups (*p* < 0.001) (Fig. 2). However, significant interactions between group and time were observed for SJC (*p* = 0.049), MDGA (*p* = 0.002), and PtGA (*p* = 0.041) indicating a significant difference with respect to change over time in these outcomes between the three CDAI trajectory groups (Fig. 2).

Application of GMM to the individual CDAI components led to degenerate groupings for 3- and 4-group models for tender joint count, patient global assessment, and physician global assessment, and degenerate groupings for 2-, 3-, and 4-group models for SJC, which indicates that the individual components do not separate the trajectories as well as when the full CDAI score is used.

The 2-group model predictions for TJC, PtGA, and MDGA did not match well with the 2-group CDAI GMM classifications, where Rapid Responders and Late Responders were combined into a single Responder group (Supplemental Table 1). When using patient global assessment alone, 69.2% of CDAI trajectory Responders are instead classified as Non-Responders. When modeling on tender joint count only, 59.1% of CDAI trajectory Responders are classified as Non-Responders. Lastly, 45.7% of CDAI trajectory Responders are classified as Non-Responders when physician global assessment is only used in the GMM model. The weighted kappa values for all 3 components are less than 0.3, indicating poor agreement between the classifications.

## Discussion

This post-hoc analysis of the ABC real-world study of patients with RA treated with subcutaneous abatacept identified three trajectory groups using CDAI scores over 12 months. Consistent with previous reports on the use of abatacept in patients with RA, patients in this study responded well to treatment [10, 19, 20]. Although patients enrolled in this study had high disease activity at baseline (mean CDAI > 30), half of patients (49%) were classified as Rapid Responders, where the average time to LDA/remission (RMST) was 5.6 months and 9.6 months to remission. Approximately one-third (38%) of patients were found to be Late Responders achieving LDA/remission within the 12-month follow-up period. Only about 14% of patients were classified as Non-Responders, and while they did not achieve LDA/remission in the 12-month period, they did show an improvement in CDAI score of about 10 points. Rapid responders had less severe disease at baseline, which differs from previous reports that showed patients with established RA who had high DAS28 responded rapidly to abatacept [11]. However, some reports have noted that abatacept retention was greater in patients with lower baseline disease [13, 14]. 

Predicting a patient’s response to bDMARD treatment is a complex challenge but highly relevant in clinical practice, and there are currently no broadly accepted predictive markers. This analysis identified lower baseline physician and patient global assessment, lower swollen joint count, higher comorbidity, and ACCP positivity, which has also been previously shown to be associated with better response and improved retention of abatacept [8, 14, 21], as well as previous bDMARD treatment as potential predictors of response to treatment over 12 months. It may also be of interest to initiate abatacept treatment in patients with preclinical RA as previous studies have found that early intervention with abatacept reduces the risk of developing RA [22, 23]. 

Current recommended practice in RA is to T2T of LDA/remission or remission. However, there is a lack of evidence and clarity with respect to the timing of changing treatment. Our results have shown that, in patients treated with abatacept, the changes in disease severity observed during the first three months are not predictive of the 12-month response. This would suggest that patients not achieving remission or LDA/remission during the first three months of treatment with abatacept could consider continuing treatment before deciding to switch.

A potential limitation of this study is that patients received follow-up visits every 3 months, which may be at longer intervals compared to those in randomized controlled trials. However, this schedule may more accurately reflect real-world settings where patients are typically followed every 3 to 6 months. There are inherent limitations to all observational studies including bias by indication and selection bias. However, the inclusion of patients for whom the physicians had already decided to treat with abatacept may reduce indication bias. Generalization of the results may be limited to patients with longstanding RA being treated with abatacept, and treatment decisions should be individualized and interpreted in this context. However, the results remain relevant to clinicians when considering initiating a biologic treatment and setting expectations for patients with RA.

One of the strengths of this study is it used a novel statistical technique combining fully-Bayesian GMM and Bayesian imputation for missing data to determine response groups over a 12-month period. This method is similar to that used in the study by Taylor et al., which utilized GMM to study CDAI response trajectories in patients with RA treated with baricitinib and found that lower baseline CDAI was associated with rapid response [16]. The method presented in the current study additionally allows for missing data to be imputed without assumptions about the distribution of the data and to select the appropriate number of classifications as determined by the best fit of the data. Finally, this study examines patient trajectories over time, which may be of more clinical relevance than assessments at specific time points.

## Conclusions

The majority of patients receiving subcutaneous abatacept had rapid or late treatment response over the 12-month period in the ABC study. Lower baseline disease severity, less comorbidity, and being biologic-naïve were associated with good response to abatacept. Among patients with established RA being treated with abatacept, treatment continuation for longer than three months may be considered even in the absence of early LDA/remission or remission to allow for optimal response and patient outcomes.

## Supplementary Information

Below is the link to the electronic supplementary material.

Supplementary Material 1

## Data Availability

Bristol Myers Squibb shares data from BMS studies that meet our data sharing criteria with qualified researchers who submit a data sharing proposal at: (https://www.bms.com/researchers-and-partners/independent-research/data-sharing-request-process.html). The data that support the findings of this study are available from the corresponding author upon reasonable request.

## Abbreviations

ABC: Abatacept best care
ACCP: Anti-cyclic citrullinated peptide
ANOVA: Analysis of variance
bDMARD: Biological disease-modifying anti-rheumatic drug
BMI: Body mass index
CDAI: Clinical disease activity index
CRP: C-reactive protein
csDMARD: Conventional synthetic disease-modifying anti-rheumatic drug
DAS: Disease activity score
DAS28: Disease activity score 28
DMARD: Disease-modifying anti-rheumatic drugs
ELPD: Expected log pointwise predictive density
ESR: Erythrocyte sedimentation rate
GMM: Growth mixture model
HAQ-DI: Health assessment questionnaire-disability index
LDA: Low disease activity
MDGA: Physician global assessment
NSAID: Non-steroidal anti-inflammatory drugs
PtGA: Patient global assessment
RA: Rheumatoid arthritis
RAPID3: Routine assessment of patient index data 3
RDCI: Rheumatic disease comorbidity index
RF: Rheumatoid factor
RMST: Restricted mean survival times
SDAI: Simple disease activity index
SJC: Swollen joint count
T2T: Treat to target
TJC: Tender joint count
tsDMARD: Targeted synthetic disease-modifying anti-rheumatic drug
WPAI: Work productivity and activity impairment
