# Supplementary material for: CDAI trajectories in patients with rheumatoid arthritis treated with abatacept in real-world
Source: Adv Rheumatol. 2026 Jun 23;66:54. doi: 10.1186/s42358-026-00554-y (PMC13543570; doi:10.1186/s42358-026-00554-y)
Supplement: Supplementary file 1 [file 42358_2026_554_MOESM1_ESM.docx]

# **Title**:

CDAI Trajectories in Patients with Rheumatoid Arthritis Treated with Abatacept in Real-World

**Authors:**

Janet E Pope^1^, John S Sampalis^2,3^, Boulos Haraoui^4^, Emmanouil Rampakakis^2,5^, Dylan Keating^2^, Fiona Allum^2^, Marc-Olivier Trepanier^6^, Louis Bessette^7^

**Affiliations:**

^1^ Divisions of Rheumatology, Epidemiology, and Biostatistics, Department of Medicine, Western University, London, ON, Canada

^2^ JSS Medical Research, Inc., Montreal, QC, Canada

^3^ Faculty of Medicine, Department of Surgery, McGill University, Université de Montréal, Montreal, QC, Canada

^4^ Institut de Rhumatologie de Montréal, Montreal, QC, Canada

^5^ Department of Pediatrics, McGill University, Montreal, QC, Canada

^6^ Scientific Advisor - Rheumatology, Bristol Myers Squibb, Montreal, QC, Canada

^7^ Université de Laval, Centre Hospitalier de l’Université Laval, Quebec, QC, Canada

# Supplemental Tables

| **Supplemental Table 1: Classifying 12-month Trajectory Groups Based on Individual CDAI Components** | | | | | |
| --- | --- | --- | --- | --- | --- |
|  | **PtGA Trajectory** | |  | **Weighted Kappa** | 0.089 |
| **CDAI Trajectory** | **Non-Responders** | **Responders** |  | **Standard error** | 0.023 |
| **Non-Responders** | 33 (94.3%) | 2 (5.7%) |  | **95% CI** | 0.044–0.134 |
| **Responders** | 153 (69.2%) | 68 (30.8%) |  |  |  |
|  | **TJC Trajectory** | |  | **Weighted Kappa** | 0.149 |
| **CDAI Trajectory** | **Non-Responders** | **Responders** |  | **Standard error** | 0.029 |
| **Non-Responders** | 34 (97.1%) | 1 (2.9%) |  | **95% CI** | 0.093–0.206 |
| **Responders** | 130 (59.1%) | 90 (40.9%) |  |  |  |
|  | **MDGA Trajectory** | |  |  |  |
| **CDAI Trajectory** | **Non-Responders** | **Responders** |  | **Weighted Kappa** | 0.211 |
| **Non-Responders** | 32 (91.4%) | 3 (8.6%) |  | **Standard error** | 0.04 |
| **Responders** | 100 (45.7%) | 119 (54.3%) |  | **95% CI** | 0.132–0.29 |
| Data are N (%). CDAI: Clinical Disease Activity Score; MDGA: Physician Global Assessment; PtGA: Patient Global Assessment; TJC: Tender Joint Count. | | | | | |
